# Targeted gene disruption in *Xenopus laevis* using CRISPR/Cas9

**DOI:** 10.1186/s13578-015-0006-1

**Published:** 2015-04-14

**Authors:** Fengqin Wang, Zhaoying Shi, Yan Cui, Xiaogang Guo, Yun-Bo Shi, Yonglong Chen

**Affiliations:** School of Life Sciences, Anhui University, Hefei, 230601 China; Department of Biology, Shenzhen Key Laboratory of Cell Microenvironment, South University of Science and Technology of China, Shenzhen, 518055 China; CAS Key Laboratory of Regenerative Biology, South China Institute for Stem Cell Biology and Regenerative Medicine, Guangzhou Institutes of Biomedicine and Health, Chinese Academy of Sciences, Guangzhou, 510530 China; Section on Molecular Morphogenesis, Program in Cellular Regulation and Metabolism, Eunice Kennedy Shriver National Institute of Child Health and Human Development (NICHD), U.S. National Institutes of Health, Bethesda, Maryland USA; University of Chinese Academy of Sciences, Beijing, 100049 China

## Dear Editor

To test if the CRISPR/Cas9 system can mediate targeted gene disruption in *Xenopus laevis*, we targeted *ptf1a/p48* and *tyrosinase* in this species and found that in addition to high sequence disruption efficiency, clear phenotypes were observed in G0 embryos. *ptf1a/p48-*targeted *X. laevis* embryos can mimic *Xenopus tropicalis ptf1a/p48-*null mutant tadpoles with respect to the loss of *pdip* expression. Simultaneous disruption of two *X. laevi*s *tyrosinase* homeologs leads to almost full albinism. Our data indicate that CRISPR/Cas9 system is a simple and efficient tool for targeted gene disruption in *X. laevis.* It can be used for phenotype analysis in G0 embryos.

Since its introduction to modern biology in the 1950s, *X. laevis* has played a central role in most disciplines of biomedical research including developmental biology, biochemistry, and molecular biology [1]. Its allotetraploid genome represents a common polyploidy in amphibians, which challenges genetic studies on this species [2]. Fortunately, by the mid 1990s, *X. tropicalis*, the only diploid species in the *Xenopus* genus, was adopted as a genetically tractable complement to the classic model *X. laevis*. Together, the two frog species provide a unique and powerful system for addressing genome duplication/evolution, functional genomics, and human disease modelling at the post-genomic era [1].

Gene knockdown in *X. laevis* has been largely dependent on antisense Morpholino oligomers since 2000, which suffers from some off-target effects [3]. A recent systematic analysis reveals poor phenotypic correlation between published Morpholino-induced morphants and mutant lines in zebrafish [4], further highlighting the off-target effects of Morpholino oligomers. Transcription activator-like effector nuclease (TALEN) was shown effective for targeted gene disruption in *X. laevis* G0 embryos [1,5-7]. With the establishment of efficient targeted gene disruption in *X. tropicalis* using the clustered regularly interspaced short palindromic repeats (CRISPR) and CRISPR-associated 9 (CRISPR/Cas9) system [8], it is worth addressing if this simple method applies to *X. laevis*.

### CRISPR/Cas9 mediated disruption of *ptf1a/p48* in *X. laevis* G0 embryos phenocopies *X. tropicalis ptf1a/p48* mutants

**Figure 1:**
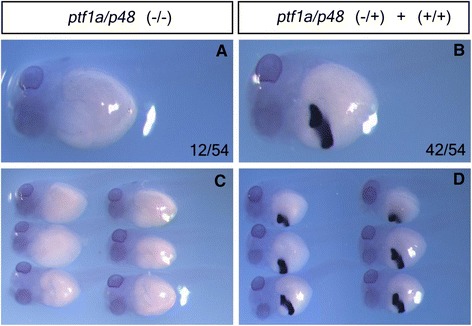
*pdip* expression is undetectable in *X. tropicalis ptf1a/p48*-null mutant tadpoles. Whole-mount in situ hybridization analysis of *pdip* expression in F2 *X. tropicalis ptf1a/p48* mutant tadpoles (stage 43). Ventral-lateral view, head to the left. (**A**, **C**) About 22% homozygous tadpoles showed complete absence of *pdip* expression. (**B**, **D**) The remainder uniformly showed strong *pdip* expression, suggesting normal pancreas development in *ptf1a/p48* heterozygous tadpoles.

The basic helix-loop-helix transcription factor Ptf1a/p48 is a key regulator early for pancreatic precursor specification and late for exocrine pancreas formation [9,10]. Our previous studies indicate that targeted disruption of *X. tropicalis ptf1a/p48* in G0 embryos using either TALENs [5] or CRISPR/Cas9 [8] leads to inhibition of pancreas formation as indicated by reduction of pancreas-specific marker gene *pdip* expression. These data are consistent with the *X. laevis* morphants [10] and *Ptf1a/p48* knockout mice [9]. Here, using our previously generated CRISPR/Cas9-targeted founder frogs, we established a *X. tropicalis ptf1a/p48* mutant line. Unlike *Ptf1a/p48-*null mutant mice that can survive whole gestation [9] likely due to mother supply of blood/nutrition and die at birth, the free-swimming *X. tropicalis ptf1a/p48* homozygous mutant tadpoles cannot develop to froglets and all died during feeding stages (around stages 47–48). Those collected at stage 43 showed complete absence of *pdip* expression (Figure 1A, C), while heterozygous tadpoles are indistinguishable from wild type siblings (Figure 1B, D), which is similar to the phenotype of *Ptf1a/p48* knockout mice [9]. The mutant phenotype obtained here also verified the reliability of previous observations on *X. laevis* morphants [10].

**Figure 2:**
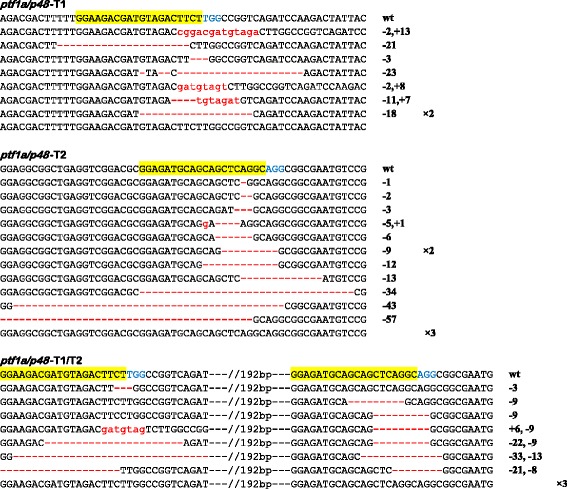
CRISPR/Cas9 is effective in targeting *X. laevis ptf1a/p48.* DNA sequencing data reveal the indel-inducing efficiencies of *ptf1a/p48*-T1 and *ptf1a/p48*-T2 in *X. laevis* embryos. For all panels, the wild-type sequence is shown at the top with the target site highlighted in yellow and the PAM sequence in blue. Red dashes indicate deletions (−) and lowercase letters in red indicate insertions (+).

Given the allotetraploid genome of *X. laevis*, every gene might have two pairs of homeologs. For *ptf1a/p48* allele, only one cDNA sequence can be found from current databases (GenBank: DQ007931.1). We designed two gRNAs targeting the first exon of this *X. laevis ptf1a/p48* locus, namely *ptf1a/p48-*T1 and *ptf1a/p48-*T2 (Additional file 1: Table S1). First we injected Cas9 mRNA together with these two gRNAs either alone or in combination into animal pole of 1-cell stage *X. laevis* embryos and assessed their indel-inducing capacity via the PCR/sequencing protocol (Additional file 1: Table S2). Based on our experience with *X. tropicalis* embryos, we set the doses of both Cas9 mRNA and gRNAs at 300 pg/embryo, which were proven optimal as on one hand efficient targeted gene disruption was obtained (Figure 2) and on the other hand no morphological malformation was observed in injected embryos. Overall, the targeted sequence disruption efficiency is above 50% (Figure 2), which is comparable to that in *X. tropicalis* [8]. It should be noted that slightly more than 1/3 of the disruption did not cause reading frame shift (Figure 2).

**Figure 3:**
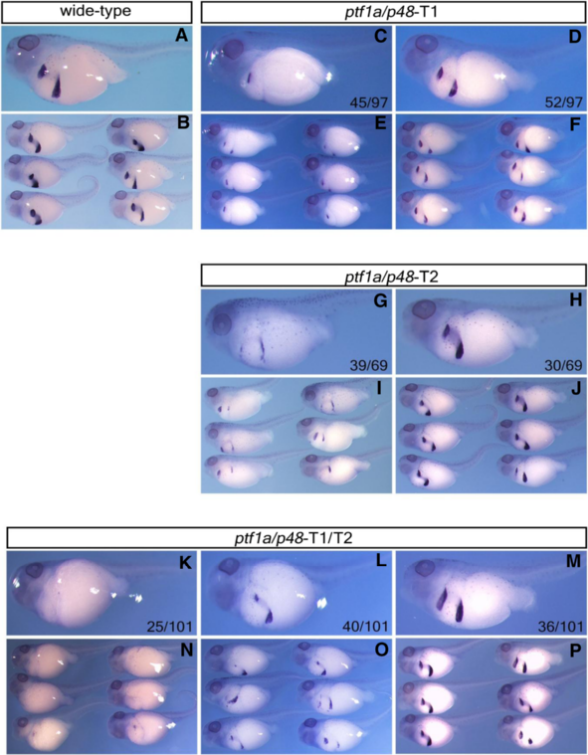
*ptf1a/p48*-targeted *X. laevis* G0 tadpoles showed reduction of *pdip* expression. Whole-mount in situ hybridization analyses of *pdip* expression (stage 42). Ventral-lateral view, head to the left. (**A**, **B**) Wild type *X. laevis* tadpoles. (**C**, **E**) Severe reduction. (**D**, **F**) Mild reduction. (**G**, **I**) Severe reduction. (**H**, **J**) Mild reduction. (**K**, **N**) Almost complete absence. (**L**, **O**) Severe reduction. (**M**, **P**) Mild reduction.

Next, we evaluated the pancreas development in injected embryos by whole mount in situ hybridization with a *pdip* probe. Similar to the observation in *ptf1a/p48-*targeted *X. tropicalis* G0 embryos [8], severe inhibition of *pdip* expression was observed in all three injection groups (Figure 3). For reasons unknown, in spite of their lower sequence disruption efficiency in comparison to the individually injected embryos (Figure 2), *ptf1a/p48-*T1 and -T2 co-injected embryos displayed stronger pancreatic phenotype with quite a few of them showing almost complete absence of *pdip* expression reminiscent of *X. tropicalis ptf1a/p48-*null mutants (Figure 3K, N and Figure 1A, C). Taken together, these data indicate that CRISPR/Cas9 system is effective in *X. laevis*. For *ptf1a/p48*, one gRNA is sufficient to cause an obvious pancreatic phenotype in *X. laevis* G0 embryos.

### Simultaneous disruption of two *X. laevis tyrosinase* homeologs leads to albinism

**Figure 4:**
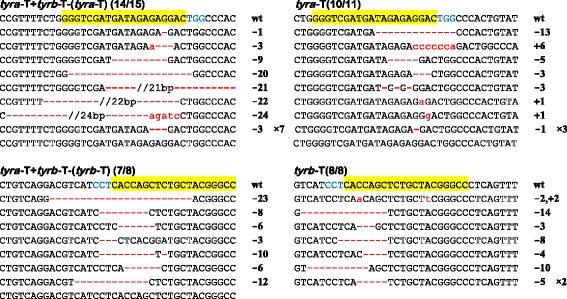
CRISPR/Cas9 is effective in targeting *X. laevis tyrosinase.* DNA sequencing data reveal the indel-inducing efficiencies of *tyra*-T and *tyrb*-T in *X. laevis* embryos. For all panels, the wild-type sequence is shown at the top with the target site highlighted in yellow and the PAM sequence in blue. Red dashes indicate deletions (−) and lowercase letters in red indicate insertions (+). The left two panels show data from *tyra*-T and *tyrb*-T co-injection and right two panels indicate individual injection results.

**Figure 5:**
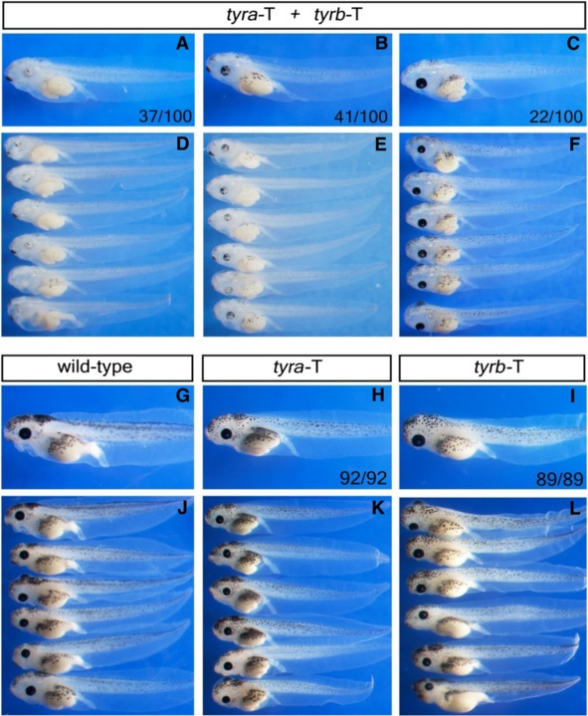
Simultaneous disruption of two *X. laevis tyrosinase* homeologs leads to albinism in G0 tadpoles (stage 42). Lateral view, head to the left. (**A**, **D**) Almost full albinism. (**B**, **E**) Severe inhibition of pigmentation. (**C**, **F**) Almost normal. (**G**, **J**) Wild type. (**H**, **I**, **K**, **L**) Almost normal.

To further test if CRISPR/Cas9 is a robust tool for gene targeting in *X. laevis*, we chose to target *tyrosinase*. There are two homeologs for *X. laevis tyrosinase*, *tyra* and *tyrb* [7]. A previous study showed that one pair of TALENs targeting a highly conserved region of the two homeologs was sufficient to induce albinism in *X. laevis* [6]. As no suitable gRNA targeting sites can be found in this conserved region for both homeologs, we have to design two gRNAs (*tyra*-T and *tyrb*-T) to target *tyra* and *tyrb*, respectively (Additional file 1: Table S1). Again, we injected Cas9 mRNA (300 pg/embryo) with these two gRNAs either alone or in combination (300 pg/embryo for each gRNA) into the animal pole of *X. laevis* fertilized eggs. PCR/sequencing data indicate that both gRNAs are very effective with targeted sequence disruption efficiencies from 87.5% up to 100% (Figure 4). The *tyra*-T and *tyrb*-T co-injected tadpoles showed severe reduction of pigmentation (Figure 5B, E), with 37% (37/100) of them showing almost full albinism (Figure 5A, D), which is stronger than TALENs-induced phenotype in *X. laevis* [6,7] and CRISPR/Cas9-induced phenotype in *X. tropicalis* [8]. An independent injection led to similar results. In contrast, although the sequence disruption efficiency remained high (Figure 4), individual injection of either *tyra*-T or *tyrb*-T did not cause any obvious alteration on embryonic pigmentation (Figure 5H, I, K, L), indicating the functional redundancy of *tyra* and *tyrb.* In sum, these data further confirm that CRISPR/Cas9 system is effective in *X. laevis.* Distinct homeologs of *X. laevis* genes can be easily disrupted by application of two gRNAs.

Due to Morpholino’s potential off-target effects, a recent study recommends mutant phenotypes as the standard metric to define gene function in zebrafish [4]. Our previous study did not detect any CRISPR/Cas9-induced off-target effects in *X. tropicalis* [8]. The issue of CRISPR/Cas9’s off-target effects in *X. laevis* remains to be defined. Nevertheless, our data demonstrate that CRISPR/Cas9 is equally a superb tool for targeted gene disruption in *X. laevis* as in *X. tropicalis*. It can be used for an immediate phenotype evaluation in *X. laevis* G0 embryos. It should be noted that the allotetraploid genome and longer generation time (1–2 years) of *X. laevis* makes it impractical to do genetic research. For establishment of stable knockout lines, we recommend to use the diploid frog *X. tropicalis* that has much shorter generation time (4–6 months).

## Additional file

Additional file 1: Table S1. Oligonucleotides used to construct gRNA expression templates. **Table S2.** PCR primers used to amplify the targeted loci.
