# Supplementary material for: Targeted gene disruption in Xenopus laevis using CRISPR/Cas9
Source: Cell Biosci. 2015 Apr 14;5:15. doi: 10.1186/s13578-015-0006-1 (PMC4403895; doi:10.1186/s13578-015-0006-1)
Supplement: Additional file 1: Table S1. — Oligonucleotides used to construct gRNA expression templates. Table S2. PCR primers used to amplify the targeted loci. [file 13578_2015_6_MOESM1_ESM.pdf]

Targeted gene disruption in *Xenopus laevis* using CRISPR/Cas9

Fengqin Wang[1,3], Zhaoying Shi[2], Yan Cui[3,5], Xiaogang Guo[3], Yun-Bo Shi[4], and Yonglong Chen[2, 3, *]

Supplements

Materials and Methods

Manipulation of *X. laevis* embryos

Wild type *X. laevis* eggs were obtained by injecting 1000 IU of human chorionic gonadotrophin into the dorsal lymph sacs of adult females 6–8 hours before egg collection. Eggs were fertilized in vitro with minced testes, dejellied with 2% cysteine hydrochloride (pH 7.8–8.0) 30 min after fertilization, and cultured in 0.1X MBS (1.76 mM NaCl, 48 mM NaHCO3, 20 mM KCl, 200 mM Hepes, 16 mM Mg2SO4, 8 mM CaCl2, 6 mM Ca(NO3)2, pH 7.4) buffer. Staging of *X. laevis* embryos was according to Nieuwkoop and Faber [1]. Cas9 mRNA (300 pg/embryo) and gRNA(s) (each 300 pg/embryo) in 5 nl volume was injected into one-cell stage embryos from the animal pole.

Generation of *Cas9* mRNA and gRNAs

Cas9 mRNA and gRNAs were generated in principle as described [2]. The oligonucleotides used for prepare gRNA templates are listed in Table S1. For gRNA transcription, the DNA templates obtained from their pUC57-T7-gRNA scaffold constructs by PCR (forward primer: 5’-GAAATTAATACGACTCACTATA-3’,

reverse primer: 5'-AAAAAAAGCACCGACTCGGTGCCAC-3') amplification were transcribed with the Transcript Aid T7 High Yield Transcription Kit (Thermo Fisher Scientific) and purified with the miRNeasy Mini Kit (Qiagen).

Establishment of *X. tropicalis ptf1a/p48* mutant line

In our previous study, we had generated *ptf1a/p48* founder frogs with CRISPR/Cas9 [2]. From one such male founder, we obtained a pool of *ptf1a/p48* heterozygous F2 frogs including males and females carrying the same genotype. Homozygous tadpoles were obtained by crossing these F2 frogs via natural mating. Several mating experiments revealed that homozygous tadpoles could not survive beyond stage 48. Some of the tadpoles at stage 43 were collected for whole mount in situ hybridization analysis.

Evaluation of gene targeting efficiency in gRNA/Cas9-injected *X. laevis* embryos

The targeting efficiency was examined at 48 hours after microinjection. We randomly collected five healthy embryos from each injection, extracted genomic DNA, amplified the targeted region by PCR (for primers see Table S2), and then cloned the purified PCR amplicons into the pMD18-T vector (Takara) by TA cloning. 10-20 single colonies were randomly picked for DNA sequencing analysis to detect any insertion or deletion (indel) mutations resulting from error-prone non-homologous end joining (NHEJ)-based repair of Cas9-created double-strand breaks. The targeting efficiency was determined by the ratio of mutant to total colonies sequenced.

Whole-mount in situ hybridization

Whole-mount in situ hybridization was performed as described [3]. The digoxigenin-labeled antisense probe for *X. laevis pdip* was prepared as described [4]. *X. tropicalis pdip* antisense probe was transcribed with T7 RNA polymerase using an RT-PCR-amplified template containing the T7 promoter (forward, 5'-GAGGAGGAGACATCAGACGA-3'; reverse, 5'-CAGTAATACGACTCACTATAGGGAATACTCAAGGACCGAAGAAA-3').

References

1. Nieuwkoop PD, Faber J. Normal table of Xenopus laevis (Daudin). A systematical and chronological survey of the development from the fertilized egg till the end of metamorphosis. Amsterdam: North-Holland Publishing Company. Guilders; 1967.
2. Guo X, Zhang T, Hu Z, Zhang Y, Shi Z, Wang Q, et al. Efficient RNA/Cas9-mediated genome editing in Xenopus tropicalis. Development. 2014;141:707-14.
3. Harland RM. In situ hybridization: an improved whole-mount method for Xenopus embryos. Methods Cell Biol. 1991;36:685-95.
4. Chen Y, Pan FC, Brandes N, Afelik S, Sölter M, Pieler T. Retinoic acid signaling is essential for pancreas development and promotes endocrine at the expense of exocrine cell differentiation in Xenopus. Dev Biol. 2004;271:144-60.

Table S1. Oligonucleotides used to construct gRNA expression templates

Target gene	Target site	PAM	Oligonucleotide 1	Oligonucleotide 2
ptf1a/p48-T1	GGAAGACGATGTAGACTTCT	TGG	ATAGGAAGACGATGTAGACTTCTGT	TAAAACAGAAGTCTACATCGTCTTC
ptf1a/p48-T2	GGAGATGCAGCAGCTCAGGC	AGG	ATAGGAGATGCAGCAGCTCAGGCGT	TAAAACGCCTGAGCTGCTGCATCTC
tyra-T	GGGTCGATGATAGAGAGGAC	TGG	ATAGGGTCGATGATAGAGAGGACGT	TAAAACGTCCTCTCTATCATCGACC
tyrb-T	CACCAGCTCTGCTACGGGCC	CCT	ATAGGCCCGTAGCAGAGCTGGTGGT	TAAAACCACCAGCTCTGCTACGGGC

Table S2. PCR primers used to amplify the targeted loci

Target loci	Forward primers (5'-3')	Reverse primers(5'-3')
ptf1a/p48-T1	TCGTTACATTAGTTTAACTGGTGCC	GTTAATGTAGCCAATGGCCAGTC
ptf1a/p48-T2	TCGTTACATTAGTTTAACTGGTGCC	GTTAATGTAGCCAATGGCCAGTC
ptf1a/p48-T1+T2	TCGTTACATTAGTTTAACTGGTGCC	GTTAATGTAGCCAATGGCCAGTC
tyra-T	CCTCCTGAGCAAGGAATGT	CACGCTGACATCCGCAAAC
tyrb-T	CAAGGAGTGTTGCCCTGTG	AGGGTTGGAGCCATTATTC
